# Growth of Global Health Spending Share in Low and Middle Income Countries

**DOI:** 10.3389/fphar.2016.00021

**Published:** 2016-02-12

**Authors:** Mihajlo Jakovljevic, Thomas E. Getzen

**Affiliations:** ^1^Faculty of Medical Sciences, University of KragujevacKragujevac, Serbia; ^2^Risk, Insurance, and Healthcare Management Department, Temple UniversityPhiladelphia, PA, USA

**Keywords:** low–middle income, developing world, third world, health expenditure, medical spending

## Historical patterns of global health spending

Over the past century medical technology has provided bold gains extending human longevity for almost several decades in most welfare economies worldwide. These public health victories came at the cost of huge increase in health spending. The USA, the largest health care market where total health expenditure (THE) grew from 4% of GDP to 15%, may serve as an example of such changes. The secular trend consisting of rising wages and incomes constitutes major factor in the rising resources dedicated to the medical care. Business cycle booms and recessions affected health care spending slowly and with a significant lag. In this sense health expenditures should not be compared to short term, quarterly or yearly fluctuations in Gross Domestic Product (GDP) but correlates well to “smoothed” income over the previous 3–6 years (Getzen, 1990).

Growth of health expenditure is driven by several underlying issues: population birth rates, per-capita income, inflation and so called “excess growth” that is mostly explained by medical technology advances or increased patient demand for services. This “excess growth” is responsible for raising the share of health care in national GDP, and thus challenging fiscal sustainability. Evidence of excess growth is seen in health insurance premiums that persistently rise faster than tax revenues or wages. Isolated excess cost growth was the key underlying reason for the surmountable surge in health care costs visible in the United States since the late 1950s. Unlike the contemporary post WWII era, previous historical records testify of stable medical costs of about 4% of GDP from 1929 to the late 1950s. U.S. Census records of employment in clinical medicine and published consumer expenditure evidence from 1850–1950 show that these costs were mostly keeping pace with wages. If they were slightly exceeding wages it was only about 0.5% annually thus it took more than a century for them to double, much slower than the quadrupling from 1960 to 2000 (Getzen, 2000). Major causes of such a sudden rise in health expenditures were huge economic development, distinctively extended longevity, control of contagious diseases, rising availability of income used to fund research in medicine, effective financing instruments, and ultimately significant discoveries in medical technologies that supported public willingness for further investment into potential novel biological drugs, implants, robotic surgery, radiation therapy, organ transplants, and other wonder technologies (Getzen, 2014).

With several decades delay, due to dissemination of knowledge and improved societal welfare across the globe, similar developments began at the far smaller scale in a large number of low and middle income world economies. Among 160 such nations in the beginning of 1990s long term trends have revealed 16 countries which made greater investments in health care and its core outcomes than most comparable nations. These countries were described by Goldman-Sachs as the world's leading emerging markets. They are listed under the acronyms BRICS (Brazil, Russia, India, China, South Africa) and Next Eleven (N-11: Bangladesh, Egypt, Indonesia, Iran, South Korea, Mexico, Nigeria, Pakistan, the Philippines, Turkey, and Vietnam). This ongoing evolution will most likely shape the appearance of global demand and supply of medical services in XXI century and we believe that therefore it deserves closer examination.

## Growth of health care spending in low and middle income countries since 1995

The last two decades have been particularly dynamic due to ending the Cold War and accelerated pace of globalization. Contemporary evolution was promising for most nations with average world THE rising from 5.7 to 6.8% GDP [a 19.3% gain or approximately 1% yearly increase over 19 years (Table 1)]. Since 1995 World Health Organization (WHO) has established and disseminated National Health Accounts (NHA) system worldwide. These efforts allowed reliable international comparison of financial flows among national health systems with diverse historical legacies. The World Bank (WB) introduced the measure of gross national income (GNI) classification of countries in 1987 with their Atlas method and GNI per capita indexed in US$ currency (World Bank Income Groups, 2015). Availability of national income per capita strongly influences health expenditure. The correlation is straightforward with a secular trend visible in long time horizons in most world regions. We applied historical lists of WB income classification to reveal patterns in global health spending. Participation of 160 low and middle income countries (as defined by WB in 1995) in global health spending (in million const. 2005 $US) was 10.7%. Nineteen years later the world was a much different place. Global welfare of nations recorded bold increases while 23 countries crossed the WB threshold for high income economies. The remaining 137 low and middle income countries (as defined by WB in 2013) were now spending 14.6% of global THE expressed in millions of constant 2005 $US. The landscape of national medical spending has evolved in favor of developing regions. The 160 countries classified as low and middle by WB in 1995 grew from 26.1% of global THE in 1995 to 39.7% in 2013. While high income economies still dominate the global landscape of medical spending, the growth of emerging economies has reduced their share of the total.

**Table 1:**
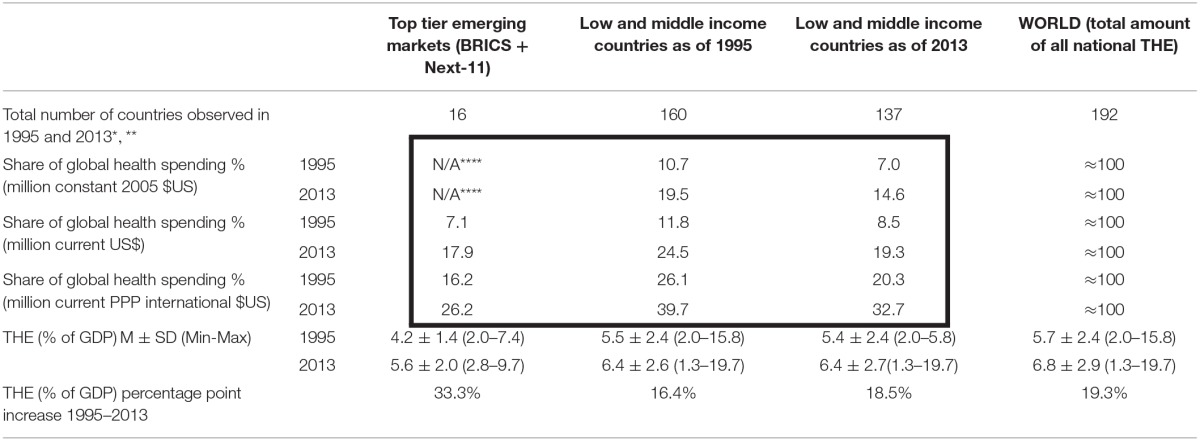
**Transformation of Global Total Health Expenditure (THE) 1995–2013**.

*Table based on WHO National Health Accounts data 1995–2013; Classification based on World Bank Historical Lists of Income level country groups 1995/2013 based on GNI per capita in US$ (Atlas methodology); Top tier Emerging Markets definition adopted based on Goldman-Sachs acronyms BRICs and Next-11*.

^*^WB Note: Income classifications are set each year on July 1 for all World Bank member economies, and all other economies with populations of more than 30,000. These official analytical classifications are fixed during the World Bank's fiscal year (ending on June 30), thus economies remain in the categories in which they are classified irrespective of any revisions to their per capita income data. The historical classifications used are as published on July 1 of each fiscal year.

^**^Total of 13 countries/legal entities were not classified according to WB Income groups while three countries ceased to exist in 1995. In 2013 there were two of such non-classified entities listed together with five countries that ceased to exist.

^***^For a total of 18 countries inclusive of Japan 2013 data are still not released officially therefore closest year available (2012 data in most cases) was used. Joint total health expenditure of these countries excluding Japan remains significantly below 1% of global THE.

^****^Among the BRICS and Next–11 emerging markets THE data expressed in terms of constant 2005 $US are lacking for Russian Federation and Pakistan for the entire 19 years long observation period and therefore inclusion of this indicator among the emerging markets was omitted entirely due to absence of data for two large nations.

## Causes of changes and leadership of BRICs + next-11 emerging nations

Jim O'Neil's grouping of BRICs was driven primarily designating those whose nominal and purchase power parity (PPP) adjusted GDP growth rates significantly outpaced those of most OECD nations before and during the worldwide economic recession. Similar ongoing development characterizes another group, identified by Goldman-Sachs' as the “Next Eleven.” Profound changes with deep and lasting impact to the global demand for and provision of healthcare services and associated expenditure have occurred. Rapid expansion of civil middle class in most of these societies has been a major underlying factor (Jakovljevic, 2015). Substantial gains in overall welfare are reflected in the expansion of health insurance coverage and diversity of medical services provided. Growth of purchasing power effectively improved affordability of advanced medical care that remains out-of-pocket expense. We witness continuing movement of global growth in health care markets from the established mature economies toward the emerging ones. Slower economic growth in most saturated high-income markets is a contributing factor. Consumer demand for medical services remains larger in traditional wealthy countries, but their share has been decreasing steadily for at least two decades.

Total amount of health care spending among BRICS and Next-11 nations became approximately six fold stronger since 1995. Share of Global Health Spending (million current US$) of these emerging nations grew almost two and a half times. This pace of development is far faster compared to that of vast majority of remaining low and middle income countries across the globe. If we observe per capita health spending it appears that general government expenditure on health and private expenditure is consistently stronger among BRICS compared to N-11. Such a historical trend was actually present prior to 1990s and spending differentials continued to exist as paths diverted even further in recent years. Out-of-pocket (OOP) expenditure on health is a significant outlier in this regard. Although both country group averages were similar at the start, N-11 OOP spending soon exceeded BRICs. These facts indicate better success rates among the BRICs in terms of reimbursement policies and insurance coverage over the past 20 years (Jakovljevic, 2014).

## Prospects for the future

Observation of health spending trends over 20 years is still insufficient to understand a “medical transformation” taking place in major national health systems worldwide. Limitations to our judgment might be imposed by reliability and comparability of large international datasets as well (Rayne, 2013). Nevertheless contemporary transformation of global health spending lays grounds for some forecasts on likely scenarios for the future. Low and middle income countries are likely to become more relevant contributor to the global health care market in the long run. Minor proportion of these countries will likely become high income economies over the next decade. Vast majority of them will continue to experience serious obstacles to the fiscal feasibility of their national health systems. Crucial challenges will remain population aging, prosperity disease and rapid urbanization leaving vulnerable rural areas. Universal health insurance coverage will still be a distant policy target for most of these governments with the notable exception of Russian Federation (Jakovljevic et al., in press). Large out of pocket expenses and informal payments will leave ordinary citizens, living close to the poverty line, vulnerable to the illness-induced catastrophic household expenditure (McIntyre et al., 2006). In some world regions with still young populations, communicable diseases control and satisfactory maternal and neonatal medical care provision shall still be a long way ahead (Barik and Thorat, 2015). Regardless of all the aforementioned weaknesses of developing world regions, it appears that most successful among these nations will become even more important players in global health arena. Heavily domination of People's Republic of China (He and Meng, 2016) followed by India in medical spending worldwide will exceed that of all other emerging markets combined. As we approach 2050 it is highly likely that financing of health care in top tier emerging nations will converge toward OECD average in terms of its effectiveness and affordability of medical care to the ordinary citizen (Jakovljevic, 2016). Major imperatives for national policy makers shall remain how to achieve universal health coverage, what services would be covered by basic insurance package and at what cost. Future research in the field should primarily be focused on key causes of out-of-pocket medical spending growth, deepening social gap among the rich and poor communities leading to health inequalities and effectiveness of contemporary policies in low and middle income countries.

## Data report methodology

Public data sources used were WHO issued Global Health Expenditure Database relying on NHA records: http://apps.who.int/nha/database/Select/Indicators/en and World Bank (WB) Income Groups; Historical country classifications based on Atlas method: http://data.worldbank.org/about/country-and-lending-groups. Filters applied to these extensive data sources were indicators referring to the national level and Global Total Health Expenditure (THE) expressed in following units: million constant 2005 $US, million current US$, million current PPP international $US and THE percentage share of national Gross Domestic Product available (GDP). Data were acquired based on reported values to the WHO and WB by the national authorities as well as independent assessments and calculations provided by WHO and WB and officially released in respective years. Readers are free to access and reuse these publicly available data at the links provided above.

## Author contributions

MJ and TG have jointly developed the research questions, study design, did all the calculations and prepared manuscript for this Data report. Therefore, they share the first authorship in this paper.

### Conflict of interest statement

The authors declare that the research was conducted in the absence of any commercial or financial relationships that could be construed as a potential conflict of interest.
